# Health Care Professionals’ Experience in a Digitally Supported Hospital-at-Home Program: Cross-Sectional Study

**DOI:** 10.2196/106615

**Published:** 2026-11-13

**Authors:** Carlos Bibiano Guillén, Eva Maria Arroyo Urea, Olga Estremera López, Sonia López Lallave, María Consolación Aranda Higuero, Cristina Ubeda García, Silvia Sánchez Sierra, Almudena Calvo Cruz, María Mir Montero

**Affiliations:** 1 Department of Emergency Medicine, Short Stay Unit and Hospital at Home Unit, Hospital Universitario Infanta Leonor, Av. Gran Vía del Este 80, Madrid, 28031, Spain, 34 635524136

**Keywords:** hospital-at-home, telemedicine, remote patient monitoring, technology-enabled care, human factors, usability, health care professionals, workload, workflow integration, care delivery models, organizational sustainability, digital health, virtual health

## Abstract

**Background:**

Hospital-at-home (HaH) programs supported by telemedicine and remote monitoring technologies are increasingly being adopted as alternatives to conventional hospitalization. Although clinical outcomes and patient satisfaction have been widely studied, less is known about health care professionals’ experiences in established, digitally supported HaH models integrated into routine clinical practice.

**Objective:**

This study aimed to assess health care professionals’ perceptions of an established, digitally supported HaH program, focusing on usability, workload, organizational integration, perceived clinical impact, and overall satisfaction.

**Methods:**

We conducted a single-center cross-sectional study in the HaH unit of Infanta Leonor University Hospital (Madrid, Spain) between January and May 2025. All health care professionals assigned to the unit were invited to participate in an anonymous electronic survey composed of 14 Likert-scale items and 1 open-ended question. The items were designed to assess understanding of the program, perceived clinical impact, interaction with technology, workload, and overall evaluation. Nonparametric tests included Mann-Whitney *U* and Kruskal-Wallis tests. *P* values from each set of 14 item-level tests were adjusted separately using the Benjamini-Hochberg procedure.

**Results:**

Of 47 invited professionals, 30 (64%) participated, including physicians (n=13, 43%), nurses (n=10, 33%), and nursing assistants (n=7, 23%). Overall perceptions of the digitally supported HaH model were generally favorable, particularly regarding perceived quality of care, perceived promotion of patient autonomy, and overall satisfaction. The largest exploratory differences across professional roles appeared in items related to the perceived contribution of technology to quality of care (*ε*²=0.46, 95% CI 0.17-0.85), recommendation of the model (*ε*²=0.45, 95% CI 0.14-0.85), and perceived promotion of patient autonomy (*ε*²=0.41, 95% CI 0.15-0.78). No significant differences were identified according to years of professional experience. Optional comments were also provided by 20% (6/30) of participants and were reviewed descriptively, offering contextual examples related to connectivity, platform functionality, and workflow challenges.

**Conclusions:**

Health care professionals generally perceived the digitally supported HaH model positively. Exploratory differences between professional groups varied by item: technology-related perceptions were less favorable among responding nurses, whereas perceived workload was higher among physicians. These findings should be interpreted cautiously, given the small subgroup sizes and the low nurse response rate. Long-term sustainability of HaH programs may benefit from role-specific organizational strategies, targeted training, and better workflow integration.

## Introduction

Hospital-at-home (HaH) care has become established in recent years as a service model that delivers hospital-level care in patients’ homes, reduces complications associated with conventional hospitalization, and eases pressure on hospital capacity [1-3]. Population aging, the rise in chronic diseases, and the need for more sustainable care models have driven the international expansion of these programs [4,5].

In parallel, the development of digital tools has profoundly transformed the organization of hospital-based home care. The incorporation of telemedicine platforms, video consultations, remote monitoring of vital signs, and digital communication systems has expanded the capacity for clinical follow-up beyond the traditional hospital setting [6,7]. These technologies may help clinicians identify patient deterioration earlier; enhance continuity of care; and support a more flexible, patient-centered approach to service delivery [8].

However, introducing digital technologies into HaH programs changes not only how care is delivered but also how professionals communicate, organize their work, and interact with patients [9,10]. Adapting to these digital environments can generate organizational benefits, but it may also introduce new cognitive demands, additional workload, and challenges related to the usability of technological tools and the integration of digital systems into routine clinical practice [11-13].

Existing evidence on HaH has focused primarily on clinical outcomes, cost reduction, and patient satisfaction [14-17]. However, the experience of health care professionals working within highly digital health care models remains less explored, particularly in established programs where telemedicine and remote monitoring are integral to daily operations. Understanding how professionals perceive the usefulness, usability, workload, and care impact of these technologies is essential to ensuring the sustainability and scalability of such programs [18-20].

In this context, the aim of this study was to evaluate health care professionals’ experience and perceptions regarding an established, digitally supported HaH model, examining aspects related to usability, organizational integration, workload, clinical communication, and perceived quality of care.

## Methods

### Study Design and Context

We conducted a single-center cross-sectional observational study in the HaH unit of Infanta Leonor University Hospital, Madrid, Spain. The study aimed to evaluate health care professionals’ perceptions and experiences regarding an established digitally supported HaH program integrated into routine clinical practice. In this study, the term “established” refers descriptively to a program implemented in 2020 that has maintained sustained operational activity and integrated digital workflows.

The evaluated model combines telemedicine consultations, remote monitoring of vital signs, structured digital symptom questionnaires, and automated clinical alerts. Since its implementation, the program has processed more than 500,000 digital alerts and currently manages approximately 1200 admissions per year. Nearly 60% of these admissions involve adults aged 65 years or older, and more than 75% of follow-up activities are conducted remotely, with in-person visits scheduled according to clinical needs.

To support this level of activity, the HaH unit is staffed by 13 physicians, 25 nurses, and 9 nursing assistants, who provide coordinated multidisciplinary care within a structured digital workflow (Figure 1). The unit manages a broad range of conditions, including respiratory tract infections, urinary tract infections, intra-abdominal infections, hematologic disorders, heart failure, skin and soft tissue infections, and osteoarticular disorders.

**Figure 1.**
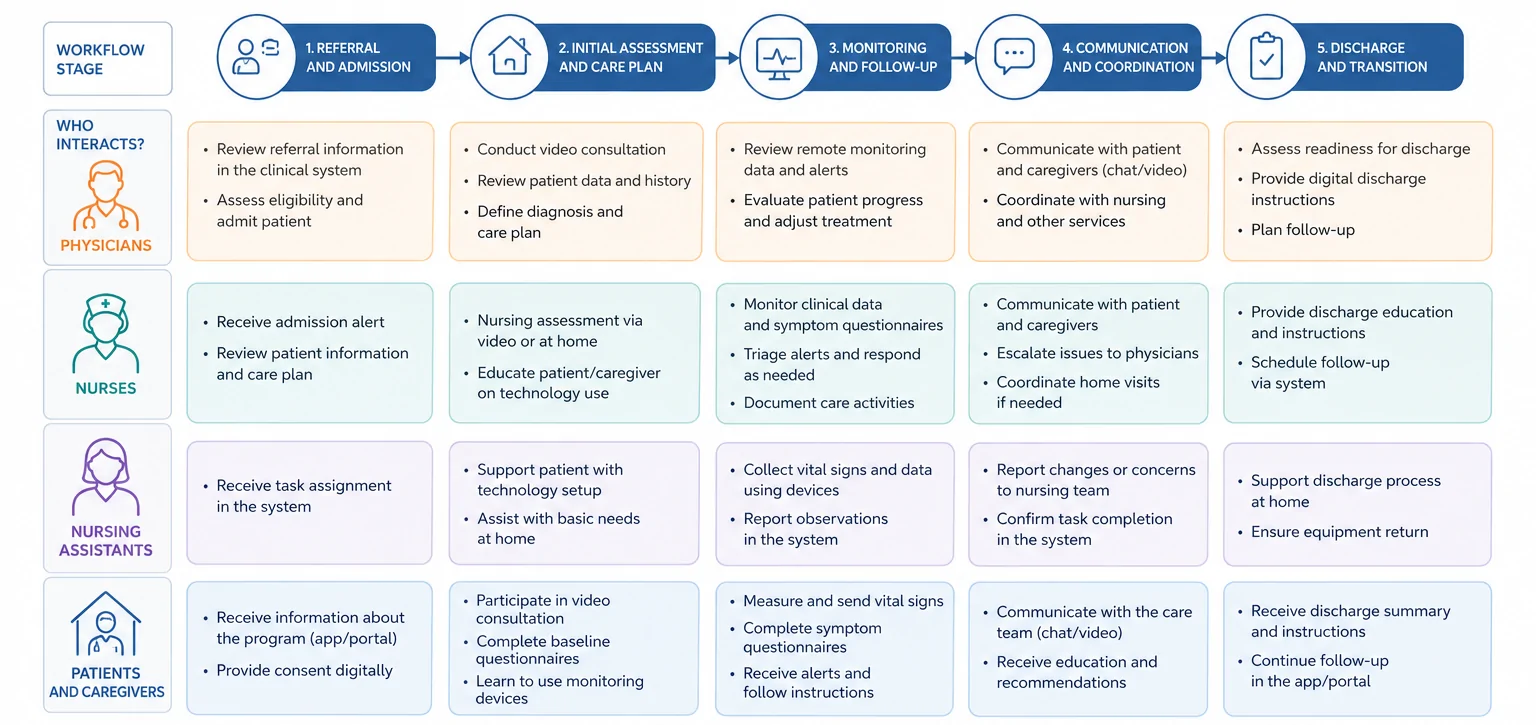
Workflow of the hospital-at-home unit, illustrating the roles and responsibilities of each professional within the digitally supported care model.

### Participants and Recruitment

Recruitment was conducted between January and May 2025 using an internally distributed QR code linked to an electronic survey. The survey was available throughout the study period, allowing participants to complete it at a time of their choice, either during or outside working hours, with no specific time window during the workday required for completion. All health care professionals assigned to the HaH unit during the study period were invited to participate (N=47).

Participation was voluntary and anonymous. Before accessing the questionnaire, participants were required to provide electronic informed consent by confirming their willingness to participate and authorizing anonymous data collection.

No additional exclusion criteria were established.

### Data Collection

In addition to survey responses, professional background variables were recorded, including professional group, years of experience, and self-reported training status related to the digitally supported HaH model. Training status was categorized as “yes,” “partially,” or “no” and reflected participants’ perceived sufficiency of training rather than objectively verified completion of a training program. A response of “yes” indicated that participants felt they had received, or were currently receiving, sufficient training to use the technological platform. “Partially” indicated that some training had been received but that additional instruction was considered necessary, and “no” indicated that no training had been received.

### Survey Instrument

A study-specific questionnaire was developed to explore health care professionals’ perceptions of an established digitally supported HaH model. The questionnaire was informed by constructs derived from the technology acceptance model [20,21] and the unified theory of acceptance and use of technology [19]. On the basis of these conceptual frameworks, the research team developed study-specific items tailored to the HaH setting to capture health care professionals’ perceptions of the digitally supported HaH model. The questionnaire covered aspects related to understanding of the program, perceived clinical impact, interaction with technology, workload, and overall evaluation of the model.

The instrument was then reviewed by 2 experts with experience in the field, who assessed the relevance and clarity of the items during a joint review meeting. On the basis of their discussion, the wording and content of the items were reviewed and agreed upon by the research team. No formal pilot test was performed before the survey was distributed.

The final instrument included 14 items rated on 5-point Likert-type scales adapted to the nature of each question (Figure S1 in Multimedia Appendix 1). Depending on the item, response anchors reflected levels of agreement, perceived quantity, satisfaction, clarity, or perceived impact. Higher scores consistently indicated more favorable evaluations, except for the workload and stress items, for which higher values denoted greater perceived burden.

Because the questionnaire included multiple related domains, different Likert response anchors, and items with different interpretive directions, no overall Cronbach α was used to support claims of psychometric reliability or validity. The instrument was treated as an exploratory, study-specific questionnaire rather than as a formal psychometric scale.

Separate domain-level reliability estimates were not considered sufficiently informative because the questionnaire included multiple related domains, some of which were represented by only 2 items. The domain structure was therefore used to organize the descriptive presentation of the results rather than to define formally validated subscales. Given the exploratory and practice-oriented nature of the study, the questionnaire was intended to capture health care professionals’ perceptions related to clinical workflows rather than function as a formal psychometric scale. Accordingly, no factor analysis was performed.

Additionally, the questionnaire included an optional open-ended question: “Would you like to add any comments or recommendations about your experience with the HaH program using technology?” Responses to this question were reviewed descriptively. No formal qualitative coding or thematic analysis was performed.

### Statistical Analysis

Categorical variables were described using absolute frequencies and percentages. Responses to Likert items were summarized using means and SDs to facilitate the overall interpretation of professional perceptions. Medians and IQRs were also calculated because of the ordinal nature of the response scales and are provided in Table S1 in Multimedia Appendix 1.

Given the small sample size, the ordinal nature of the Likert scales, and the presence of skewed distributions with ceiling effects in several items, nonparametric tests were used for group comparisons. Differences according to years of professional experience were analyzed using the Mann-Whitney *U* test, comparing professionals with fewer than 10 years of experience with those with 10 or more years. This dichotomization was selected based on the observed distribution of the sample and to avoid creating smaller subgroups that could limit the stability and interpretability of comparisons. Differences between professional groups were evaluated using the Kruskal-Wallis test.

*P* values from the Mann-Whitney *U* and Kruskal-Wallis tests were adjusted separately across the 14 items using the Benjamini-Hochberg false discovery rate procedure. The 2 correction families were treated separately because they addressed distinct grouping variables (years of professional experience and professional group), while the same 14 item-level outcomes were evaluated within each family. Within each family, the Benjamini-Hochberg correction was applied across all 14 items because these were the prespecified outcomes evaluated for each grouping variable. This approach preserved the distinction between the 2 grouping comparisons while controlling the false discovery rate across the 14 outcomes within each family. An adjusted *P* value of <.05 was considered statistically significant. Raw *P* values for both sets of comparisons are provided in Table S1 in Multimedia Appendix 1.

For items with Kruskal-Wallis *P* values that remained significant after adjustment, the Dunn multiple-comparisons test was performed to identify pairwise differences. GraphPad Prism (version 8.0.1; GraphPad Software Inc) reported multiplicity-adjusted *P* values for the pairwise comparisons; no additional multiplicity correction was applied to these adjusted *P* values.

To quantify the magnitude of these group differences, effect sizes were calculated using *ε*² for Kruskal-Wallis analyses and *r* for Mann-Whitney *U* tests, according to the standard methodology for nonparametric group comparisons [22,23]. For the latter, positive values indicated higher scores among professionals with fewer than 10 years of experience, whereas negative values indicated higher scores among those with 10 or more years of experience. The 95% CIs for both effect size measures were estimated using a percentile bootstrap resampling approach with 10,000 iterations.

Statistical analyses were performed using Python (version 3.13.0; Python Software Foundation) and GraphPad Prism (version 8.0.1).

### Ethical Considerations

The study was approved by the ethics committee of the Infanta Leonor University Hospital (56301711). Participation was voluntary and anonymous. All participants provided electronic informed consent before completing the questionnaire. Data were treated confidentially and analyzed in accordance with current data protection regulations.

## Results

### Participant Characteristics

A total of 30 health care professionals from the HaH unit participated in the study, corresponding to 30 (64%) of 47 invited professionals. The sample included physicians (n=13, 43%), nurses (n=10, 33%), and nursing assistants (n=7, 23%). Participation varied across professional groups: all physicians completed the survey (13/13, 100%), followed by nursing assistants (7/9, 78%) and nurses (10/25, 40%). Slightly more than half of the participants (17/30, 57%) had fewer than 10 years of professional experience. Of the 30 participants, 22 (73%) reported sufficient training on the digital platform, 6 (20%) reported partial training, and 2 (7%) reported no training (Table 1).

**Table 1.** Characteristics of participating health care professionals (n=30).

Characteristics	Participants, n (%)
Professional group	
Physicians	13 (43)
Nurses	10 (33)
Nursing assistants	7 (23)
Years of experience	
<10 years	17 (57)
*≥*10 years	13 (43)
Training on the HaH^a^ unit’s technological platform (completed or ongoing)	
Yes	22 (73)
Partially	6 (20)
No	2 (7)

a HaH: hospital-at-home.

### Survey Results

Overall perceptions of the program were positive (Figure 2). The highest scores were observed in items related to understanding of the program, perceived quality of care, and recommendation of the model. In contrast, perceived stress associated with HaH-related work received the lowest ratings. Greater variability was observed in responses related to ease of technology use, workload, and adequacy of technical support, suggesting more heterogeneous experiences across participants.

**Figure 2.**
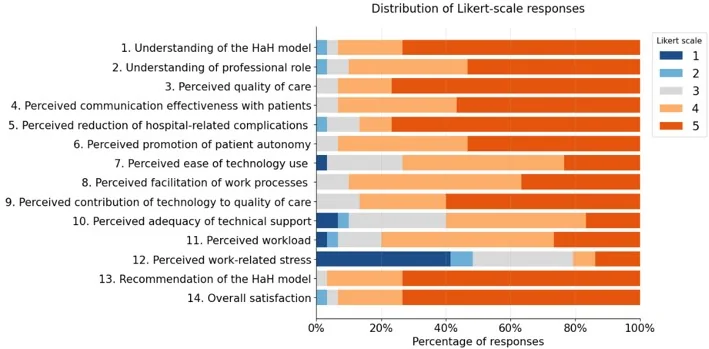
Distribution of 5-point Likert scale responses (percentages per item). For workload and stress, higher scores indicate greater perceived burden; for all other items, higher scores indicate more favorable evaluations. HaH: hospital-at-home.

Additionally, no statistically significant differences were identified according to years of professional experience after Benjamini-Hochberg adjustment (Table S2 in Multimedia Appendix 1). However, several exploratory differences across professional groups were observed (Table 2). Dunn post hoc comparisons indicated that significant pairwise differences occurred mainly between physicians and responding nurses, with nurses reporting lower scores in perceived communication effectiveness with patients (item 4), perceived promotion of patient autonomy (item 6), ease of technology use (item 7), facilitation of work processes (item 8), perceived contribution of technology to quality of care (item 9), and recommendation of the HaH model (item 13; Table S3 in Multimedia Appendix 1). Perceived workload (item 11) showed a different pattern, with physicians reporting higher scores, reflecting greater perceived burden.

**Table 2.** Mean (SD) scores of the questionnaire items according to professional groups (Likert scale 1‐5)^a^.

Conceptual category and item		Physicians, mean (SD)	Nurses, mean (SD)	Nursing assistants, mean (SD)	BH-adjusted^b^ *P* value^c^	Effect size, ε² (95% CI)^d^
Understanding of the program						
Understanding of the HaH^e^ model		4.6 (0.7)	4.7 (0.5)	4.6 (1.1)	.82	0 (0‐0.19)
Understanding of professional role		4.6 (0.7)	4.2 (0.6)	4.3 (1.1)	.31	0.03 (0‐0.41)
Perceived clinical impact						
Perceived quality of care		4.8 (0.4)	4.4 (0.8)	5 (0)	.19	0.07 (0‐0.39)
Perceived communication effectiveness with patients		4.7 (0.5)	4.0 (0.7)	4.9 (0.4)	*.03*	0.28 (0.02‐0.67)
Perceived reduction of hospital-related complications		4.8 (0.6)	4.1 (1.1)	5 (0)	.07	0.17 (0‐0.57)
Perceived promotion of patient autonomy		4.9 (0.4)	3.9 (0.6)	4.6 (0.5)	*.007*	0.41 (0.15‐0.78)
Interaction with technology						
Perceived ease of technology use		4.3 (0.6)	3.3 (1.0)	4.0 (0.8)	*.049*	0.20 (0.02‐0.52)
Perceived facilitation of work processes		4.5 (0.5)	3.8 (0.6)	4.4 (0.5)	*.049*	0.20 (0‐0.53)
Perceived contribution of technology to quality of care		4.8 (0.4)	3.8 (0.6)	4.7 (0.8)	*.006*	0.46 (0.17‐0.85)
Perceived adequacy of technical support		4.0 (0.4)	3.1 (1.2)	3.6 (1.4)	.11	0.12 (0‐0.60)
Workload						
Perceived workload		4.4 (1.1)	3.7 (0.7)	3.6 (0.5)	*.02*	0.31 (0.02‐0.71)
Perceived work-related stress		2.8 (1.8)	2.2 (1.0)	2.1 (1.2)	.79	0 (0‐0.25)
Overall evaluation						
Recommendation of the HaH model		4.9 (0.3)	4.2 (0.6)	5 (0)	*.006*	0.45 (0.14‐0.85)
Overall satisfaction		4.7 (0.9)	4.4 (0.7)	4.9 (0.4)	.21	0.06 (0‐0.46)

a Higher scores indicate higher levels of the construct measured; for workload and stress items, higher scores indicate greater perceived burden.

b BH: Benjamini-Hochberg.

c Kruskal-Wallis test. *P* values were adjusted using the BH false discovery rate correction across the 14 questionnaire items. BH adjustment was performed using the unrounded raw *P* values. Adjusted *P*<.05 are shown in italics.

d Effect sizes (ε²) were reported with 95% CIs and interpreted as small (ε²≥0.01), moderate (ε²≥0.08), or large (ε²≥0.26). The 95% CIs were estimated using percentile bootstrap resampling with 10,000 iterations.

e HaH: hospital-at-home.

An exploratory descriptive analysis was also conducted to examine responses to technology-related items according to self-reported training status (Table S4 in Multimedia Appendix 1). Participants reporting sufficient training (“yes”) had descriptively higher ratings for ease of technology use and for the perceived contribution of technology to the quality of care, whereas those indicating partial or no training showed lower or more variable scores. Perceived adequacy of technical support displayed a similar descriptive pattern. In contrast, ratings related to the perceived facilitation of work processes appeared relatively consistent across the 3 training categories. Because the no-training group included only 2 participants, these comparisons are descriptive and were not subjected to inferential testing.

Of the 30 participants, 6 (20%) provided optional comments. Given the small number of responses, these comments were reviewed descriptively and are presented as supplementary contextual information (Table S5 in Multimedia Appendix 1). These comments offered contextual examples related to connectivity, platform functionality, and workflow challenges.

## Discussion

### Principal Findings and Comparison With Prior Work

This study evaluated health care professionals’ perceptions and experiences of an established digitally supported HaH program, with particular attention to the interaction between technology and the organization of clinical work in a unit where telemedicine, remote monitoring, and automated alert systems are integrated into routine care workflows.

Overall, professionals reported a favorable perception of the model, particularly regarding perceived clinical usefulness, perceived quality of care, and overall satisfaction with their experience in the unit. These findings are consistent with previous studies describing high levels of professional acceptance associated with the use of digital tools in health care settings [24,25]. However, unlike many previous investigations focused on pilot implementations or emergency deployment during the COVID-19 pandemic, our study evaluated an established model integrated into everyday clinical practice. This context allows a more realistic assessment of how sustained digital integration is perceived by professionals, while not providing evidence of objective clinical outcomes.

A key exploratory finding was that several differences between professional groups remained statistically significant after adjustment, especially between physicians and responding nurses, in aspects related to interaction with technology, perceived workload, and perceived contribution of technology to quality of care. The direction of these differences varied by item: responding nurses reported lower scores on several technology-related items, whereas physicians reported higher perceived workload. This result aligns with prior research showing that the experience of using digital tools may vary according to clinical role and the type of interaction each professional maintains with the technology throughout the care process [12,26-28]. However, these results should be interpreted with caution given the small subgroup sizes and the potential for nonresponse bias, particularly among nurses, who had the lowest participation rate (10/25, 40%). Because no information was available on the characteristics or perceptions of nonresponding nurses, it remains unclear whether those with more positive or negative experiences were more likely to participate.

In our setting, the observed differences may be related to the organizational structure of the digitally supported HaH model. Nursing staff interact more frequently with the digital platform due to responsibilities related to remote monitoring, alert management, and structured documentation of clinical information. In contrast, physicians use the system in a more targeted manner, primarily for clinical assessment and decision-making. This difference in the intensity and frequency of use may help explain the observed disparities, although the cross-sectional design does not establish causation.

The integration of automated monitoring systems and clinical alerts constitutes another relevant aspect of digitally supported HaH models. Although these tools are intended to facilitate early detection of clinical deterioration, they may also contribute to alert fatigue and increased cognitive workload when alerts are frequent, insufficiently prioritized, or poorly integrated into existing workflows [18,29].

Importantly, despite the observed differences in workflow-related factors, no significant differences were identified regarding overall satisfaction or work-related stress. This finding suggests that health care professionals may perceive the digitally supported HaH model as clinically valuable despite experiencing challenges associated with digital care delivery. Previous studies have shown that professional acceptance depends not only on usability but also on organizational factors and the clinical value that professionals attribute to the system [21,30].

These findings have several practical implications for the implementation of digitally supported HaH programs. First, the implementation of digital tools should be accompanied by organizational adaptation strategies and tailored training according to professional roles. Although formal comparisons could not be conducted due to small subgroup sizes, the exploratory descriptive analysis showed that participants who reported sufficient training had higher descriptive ratings for ease of technology use and perceived adequacy of technical support. In this context, nurse-centered interface design, clearer alert prioritization, simplified documentation workflows, and protected time for remote monitoring may help address the usability and workflow integration needs identified in this study. Second, improving interoperability between digital platforms and clinical information systems may be important for reducing administrative burden and avoiding duplication of tasks.

Furthermore, the availability of fast and accessible technical support could help alleviate part of the workload associated with remote care. Finally, HaH programs should systematically incorporate the evaluation of professional experience as a relevant indicator of organizational quality and model sustainability. Beyond clinical outcomes alone, long-term adoption of HaH programs may depend on how well digital tools fit into everyday clinical workflows.

### Limitations

This study has several limitations. First, the sample size was relatively small, and subgroup sizes were particularly limited (13 physicians, 10 nurses, and 7 nursing assistants), which may limit statistical power and reduce generalizability. In particular, the exploratory analysis according to training status was limited by the marked imbalance between categories, particularly the very small no-training group (n=2), and was therefore restricted to descriptive analysis. Second, the low nurse response rate (10/25, 40%) raises the possibility of nonresponse bias. Although the survey was available throughout the study period and could be completed at a time of the participants’ choice, workload may still have influenced participation, and nurses with particularly positive or negative experiences may have been more likely to respond.

Third, the questionnaire was study specific and was not formally validated as a psychometric instrument. Although it was informed by established frameworks and reviewed by experts, no psychometric validity or reliability claim is made on the basis of the questionnaire, and no factor analysis was performed.

Fourth, while the digitally supported HaH program has been established since 2020, its operational characterization has been limited to the indicators available for this study. More granular measures, such as episode duration, the number of remote contacts or in-person visits per admission, and alert volume per patient or episode, were not available but could provide additional context on program operations.

Finally, the open-ended component was limited because only a small number of participants provided open-ended responses. As this study was conducted in a single established HaH program, findings may not be directly transferable to recently implemented programs, programs with different staffing models, or settings using different digital platforms.

### Conclusions

Health care professionals in this single-center established HaH program reported generally positive perceptions of the digitally supported model, although exploratory differences in usability, workflow-related perceptions, and perceived workload varied by professional role. Exploratory findings suggest that sustaining digitally supported HaH programs over time may benefit from role-specific support, workflow adaptation, and continued organizational investment. In particular, nurse-centered interface design, alert prioritization, documentation simplification, protected time for remote monitoring, and timely technical support may warrant consideration. Future multicenter studies with larger samples and validated instruments are needed to determine whether such strategies effectively enhance professional experience and workflow integration.

## Supplementary material

10.2196/106615 Multimedia Appendix 1 Survey questionnaire administered to health care professionals participating in the hospital-at-home program.

## Abbreviations

HaH: hospital-at-home

## References

[1] Wong HL, Ong CY, Ngo HJ, Yeo SS, Lee MH (2024). An overview of hospital-at-home versus other models of care. J Gen Fam Med.

[2] Romero-Ruperto S, Llaneras Artigues J, Mosquera-Brea M, Jiménez-Moreno FX (2025). Hospitalización a domicilio en la asistencia de los pacientes con enfermedad aguda [Hospitalization at home in the assistance for patients with acute pathology] [Article in English, Spanish]. Med Clin (Barc).

[3] Sultani K, Smeulers M, de Vries R, Zonderhuis BM, Nanayakkara PW (2025). Transforming acute care: a scoping review on the effectiveness, safety and implementation challenges of hospital-at-home models. BMJ Open.

[4] Leong MQ, Lim CW, Lai YF (2021). Comparison of hospital-at-home models: a systematic review of reviews. BMJ Open.

[5] Ko SQ, Goh J, Tay YK (2022). Treating acutely ill patients at home: data from Singapore. Ann Acad Med Singap.

[6] Vudathaneni VK, Lanke RB, Mudaliyar MC, Movva KV, Mounika Kalluri L, Boyapati R (2024). The impact of telemedicine and remote patient monitoring on healthcare delivery: a comprehensive evaluation. Cureus.

[7] Snoswell CL, Chelberg G, De Guzman KR (2023). The clinical effectiveness of telehealth: a systematic review of meta-analyses from 2010 to 2019. J Telemed Telecare.

[8] Adirim T, Adirim T (2025). Digital Health, AI and Generative AI in Healthcare.

[9] Isakov TM, Härkönen H, Atkova I (2024). From challenges to opportunities: digital transformation in hospital-at-home care. Int J Med Inform.

[10] Cresswell K, Sheikh A (2013). Organizational issues in the implementation and adoption of health information technology innovations: an interpretative review. Int J Med Inform.

[11] Kaihlanen AM, Laukka E, Nadav J (2023). The effects of digitalisation on health and social care work: a qualitative descriptive study of the perceptions of professionals and managers. BMC Health Serv Res.

[12] Low SY, Ko SQ, Ang IY (2025). Health care providers’ experiences and perceptions with telehealth tools in a hospital-at-home program: mixed methods study. JMIR Hum Factors.

[13] Mohammadnejad F, Freeman S, Klassen-Ross T, Hemingway D, Banner D (2023). Impacts of technology use on the workload of registered nurses: a scoping review. J Rehabil Assist Technol Eng.

[14] de Sousa Vale J, Franco AI, Oliveira CV, Araújo I, Sousa D (2020). Hospital at home: an overview of literature. Home Health Care Manag Pract.

[15] Nikmanesh P, Arabloo J, Gorji HA (2024). Dimensions and components of hospital-at-home care: a systematic review. BMC Health Serv Res.

[16] Shaw A, Moodley M, McSporran K (2021). P18 Safety and effectiveness of an integrated, telehealth-led supported discharge service for Covid-19. Thorax.

[17] Ko SQ, Chua CM, Koh SH, Lim YW, Shorey S (2023). Experiences of patients and their caregivers admitted to a hospital-at-home program in Singapore: a descriptive qualitative study. J Gen Intern Med.

[18] Greenhalgh T, Wherton J, Papoutsi C (2017). Beyond adoption: a new framework for theorizing and evaluating nonadoption, abandonment, and challenges to the scale-up, spread, and sustainability of health and care technologies. J Med Internet Res.

[19] Venkatesh V, Thong JY, Xu X (2016). Unified theory of acceptance and use of technology: a synthesis and the road ahead. J Assoc Inf Syst.

[20] Holden RJ, Karsh BT (2010). The technology acceptance model: its past and its future in health care. J Biomed Inform.

[21] Gagnon MP, Orruño E, Asua J, Abdeljelil AB, Emparanza J (2012). Using a modified technology acceptance model to evaluate healthcare professionals’ adoption of a new telemonitoring system. Telemed J E Health.

[22] Cohen J (1988). Statistical Power Analysis for the Behavioral Sciences.

[23] Fiel Peres F (2026). Effect sizes for nonparametric tests. Biochem Med (Zagreb).

[24] Lampickienė I, Davoody N (2022). Healthcare professionals’ experience of performing digital care visits-a scoping review. Life (Basel).

[25] Tan AJ, Rusli KD, McKenna L, Tan LL, Liaw SY (2024). Telemedicine experiences and perspectives of healthcare providers in long-term care: a scoping review. J Telemed Telecare.

[26] Frennert S, Petersson L, Erlingsdottir G (2023). “More” work for nurses: the ironies of eHealth. BMC Health Serv Res.

[27] Turner K, Bobonis Babilonia M, Naso C (2022). Health care providers’ and professionals’ experiences with telehealth oncology implementation during the COVID-19 pandemic: a qualitative study. J Med Internet Res.

[28] MacNeill V, Sanders C, Fitzpatrick R (2014). Experiences of front-line health professionals in the delivery of telehealth: a qualitative study. Br J Gen Pract.

[29] Michels EA, Gilbert S, Koval I, Wekenborg MK (2025). Alarm fatigue in healthcare: a scoping review of definitions, influencing factors, and mitigation strategies. BMC Nurs.

[30] Cruz-Cunha MM, Miranda IM, Gonçalves P (2013). Handbook of Research on ICTs and Management Systems for Improving Efficiency in Healthcare and Social Care.

