# Supplementary material for: Health Care Professionals’ Experience in a Digitally Supported Hospital-at-Home Program: Cross-Sectional Study
Source: JMIR Hum Factors. 2026 Nov 13;13:e106615. doi: 10.2196/106615 (PMC13766580; doi:10.2196/106615)
Supplement: Supplementary file 1 [file humanfactors-v13-e106615-s001.docx]

Principio del formulario

| **Do you consent to participate voluntarily in this survey? By selecting 'Yes', you voluntarily consent to participate and authorize the anonymous collection and analysis of your survey responses.**  ⃝ Yes, I consent and wish to participate ⃝ No, I do not consent  **Type of professional** ⃝ Physician ⃝ Nurse ⃝ Nursing assistant **Years of professional experience** ⃝ <1 year ⃝ 1-5 years ⃝ 6-10 years ⃝ >10 years **Have you received or are you currently receiving sufficient training on the HaH digital platform?**  ⃝ Yes ⃝ Partially ⃝ No **Please rate each question using a 1‑to‑5 scale, where 1 represents the lowest value (dissatisfaction, disagreement, or low quantity) and 5 represents the highest value (satisfaction, agreement, or high quantity), as applicable.** | |
| --- | --- |
| *Understanding of the program* | **1. Were you clearly informed about the HaH program?**  ⃝ 1 (Not at all clearly) ⃝ 2 ⃝ 3 ⃝ 4 ⃝ 5 (Very clearly) |
|  | **2. Were your tasks and role within the HaH program clearly explained to you?**  ⃝ 1 (Not at all clearly) ⃝ 2 ⃝ 3 ⃝ 4 ⃝ 5 (Very clearly) |
| *Perceived clinical impact* | **3. Do you consider that the HaH model enables the provision of high-quality care?**  ⃝ 1 (Strongly disagree) ⃝ 2 ⃝ 3 ⃝ 4 ⃝ 5 (Strongly agree) |
|  | **4. Is overall communication with patients effective (video calls, phone calls, or in-person interactions)?**  ⃝ 1 (Not at all effective) ⃝ 2 ⃝ 3 ⃝ 4 ⃝ 5 (Very effective) |
|  | **5. Do you consider that the HaH model has reduced hospital-related complications?**  ⃝ 1 (Strongly disagree) ⃝ 2 ⃝ 3 ⃝ 4 ⃝ 5 (Strongly agree) |
|  | **6. The HaH program promotes patient autonomy in managing their own care.**  ⃝ 1 (Strongly disagree) ⃝ 2 ⃝ 3 ⃝ 4 ⃝ 5 (Strongly agree) |
| *Interaction with technology* | **7. Rate the ease of use of the technology platform used in the HaH program.**  ⃝ 1 (Very difficult) ⃝ 2 ⃝ 3 ⃝ 4 ⃝ 5 (Very easy) |
|  | **8. Do you think that the use of technology (platform, video calls, tablet, app, sensors, etc.) facilitates your work?**  ⃝ 1 (Strongly disagree) ⃝ 2 ⃝ 3 ⃝ 4 ⃝ 5 (Strongly agree) |
|  | **9. The technology used in the HaH program improves the quality of patient care (monitoring, clinical attention, and treatment).**  ⃝ 1 (Strongly disagree) ⃝ 2 ⃝ 3 ⃝ 4 ⃝ 5 (Strongly agree) |
|  | **10. Was the technical support provided by the professional team sufficient to resolve issues with the technology (apps, tablet, platform) promptly?**  ⃝ 1 (Completely insufficient) ⃝ 2 ⃝ 3 ⃝ 4 ⃝ 5 (Completely sufficient) |
| *Workload* | **11. Rate your workload in the HaH program.**  ⃝ 1 (Very low) ⃝ 2 ⃝ 3 ⃝ 4 ⃝ 5 (Very high) |
|  | **12. Working with patients at home generates additional stress or concern for me.**  ⃝ 1 (Strongly disagree) ⃝ 2 ⃝ 3 ⃝ 4 ⃝ 5 (Strongly agree) |
| *Overall evaluation* | **13. I would recommend the digitally supported HaH model to other healthcare professionals.**  ⃝ 1 (Strongly disagree) ⃝ 2 ⃝ 3 ⃝ 4 ⃝ 5 (Strongly agree) |
|  | **14. Are you satisfied with your overall experience in the HaH program?**  ⃝ 1 (Very dissatisfied) ⃝ 2 ⃝ 3 ⃝ 4 ⃝ 5 (Very satisfied) |
| **Would you like to add any comments or recommendations about your experience with the HaH program using technology?** | |

Final del formularioFigure S1. Survey instrument.

Table S1. Median [IQR] scores of questionnaire items according to years of professional experience and professional groups (Likert scale 1–5). ^a^ Comparisons according to years of professional experience were performed using the Mann–Whitney U test. ^b^ Comparisons across professional groups were performed using the Kruskal–Wallis test. Raw *P* values are reported for the 14 item-level comparisons within each analysis. Raw *P* values are reported to two decimal places, except for values <.01, which are reported to three decimal places.

| Conceptual category | Item | <10 years, median [IQR] | ≥10 years, median [IQR] | *Raw P value ^a^* | Physicians, median [IQR] | Nurses, median  [IQR] | Nursing assistants, median [IQR] | *Raw P value ^b^* |
| --- | --- | --- | --- | --- | --- | --- | --- | --- |
| *Understanding of the program* | 1. Understanding of the HaH model | 5 [5-5] | 5 [4-5] | P=.35 | 5 [4-5] | 5 [4-5] | 5 [5-5] | P=.83 |
|  | 2. Understanding of professional role | 5 [5-5] | 4 [4-5] | P=.01 | 5 [4-5] | 4 [4-5] | 5 [4-5] | P=.26 |
| *Perceived clinical impact* | 3. Perceived quality of care | 5 [5-5] | 5 [4-5] | P=.37 | 5 [4.5-5] | 5 [3.75-5] | 5 [5-5] | P=.14 |
|  | 4. Communication effectiveness with patients | 5 [4-5] | 4 [4-5] | P=.20 | 5 [4-5] | 4 [3.75-4.25] | 5 [5-5] | P=.01 |
|  | 5. Reduction of hospital-related complications | 5 [5-5] | 5 [4-5] | P=.39 | 5 [5-5] | 4.5 [3-5] | 5 [5-5] | P=.04 |
|  | 6. Promotion of patient autonomy | 5 [4-5] | 4 [4-5] | P=.64 | 5 [5-5] | 4 [3.75-4] | 5 [4-5] | P=.001 |
| *Interaction with technology* | 7. Ease of technology use | 4 [4-4.5] | 4 [3-4.5] | P=.49 | 4 [4-5] | 3.5 [3-4] | 4 [3-5] | P=.02 |
|  | 8. Facilitation of work processes | 4 [4-5] | 4 [4-5] | P=.50 | 5 [4-5] | 4 [3-4] | 4 [4-5] | P=.02 |
|  | 9. Perceived contribution of technology to quality of care | 5 [4-5] | 5 [4-5] | P=.37 | 5 [5-5] | 4 [3-4] | 5 [5-5] | P=.001 |
|  | 10. Adequacy of technical support | 4 [4-5] | 3 [3-4] | P=.01 | 4 [4-4] | 3 [2.75-3.5] | 4 [3-5] | P=.07 |
| *Workload* | 11. Perceived workload | 4 [4-5] | 4 [3.5-4] | P=.10 | 5 [4-5] | 4 [3.75-4] | 4 [3-4] | P=.005 |
|  | 12. Work-related stress | 1 [1-3] | 3 [1.25-4.75] | P=.05 | 3 [1-5] | 3 [1-3] | 2 [1-3] | P=.74 |
| *Overall evaluation* | 13. Recommendation of the HaH model | 5 [4-5] | 5 [5-5] | P=.31 | 5 [5-5] | 4 [4-5] | 5 [5-5] | P=.001 |
|  | 14. Overall satisfaction | 5 [5-5] | 5 [4-5] | P=.39 | 4.5 [4-5] | 5 [5-5] | 5 [5-5] | P=.16 |

Table S2. Mean (SD) scores of questionnaire items according to years of professional experience (Likert scale 1–5). Higher scores indicate higher levels of the construct measured; for workload and stress items, higher scores indicate greater perceived burden. ᵃ Mann–Whitney U test. *P* values were adjusted using the Benjamini–Hochberg (BH) False Discovery Rate correction across the 14 questionnaire items. Benjamini–Hochberg adjustment was performed using the unrounded raw *P* values. Adjusted P values <.05 are shown in bold. ^b^ Effect size with 95% confidence intervals (95% CI), r ≥ 0.10 small, ≥ 0.30 moderate, ≥ 0.50 large. The 95% CIs were estimated using percentile bootstrap resampling with 10,000 iterations.

| Conceptual category | Item | <10 years, mean (SD) | ≥10 years, mean (SD) | *BH-adjusted P* value ^a^ | Effect size, r (95% CI) ^b^ |
| --- | --- | --- | --- | --- | --- |
| *Understanding of the program* | 1. Understanding of the HaH model | 4.8 (0.6) | 4.5 (0.8) | *P*=.51 | 0.16  (-0.11 to 0.40) |
|  | 2. Understanding of professional role | 4.8 (0.6) | 4.1 (0.8) | *P*=.09 | 0.44  (0.14 to 0.69) |
| *Perceived clinical impact* | 3. Perceived quality of care | 4.8 (0.4) | 4.6 (0.7) | *P*=.51 | 0.13  (-0.11 to 0.38) |
|  | 4. Perceived communication effectiveness with patients | 4.7 (0.5) | 4.4 (0.7) | *P*=.51 | 0.22  (-0.08 to 0.49) |
|  | 5. Perceived reduction of hospital-related complications | 4.8 (0.6) | 4.5 (0.9) | *P*=.51 | 0.13  (-0.13 to 0.37) |
|  | 6. Perceived promotion of patient autonomy | 4.5 (0.7) | 4.4 (0.6) | *P*=.64 | 0.11  (-0.21 to 0.42) |
| *Interaction with technology* | 7. Perceived ease of technology use | 4.1 (0.6) | 3.8 (1.0) | *P*=.54 | 0.13  (-0.20 to 0.44) |
|  | 8. Perceived facilitation of work processes | 4.2 (0.7) | 4.4 (0.6) | *P*=.54 | -0.13  (-0.44 to 0.19) |
|  | 9. Perceived contribution of technology to quality of care | 4.6 (0.7) | 4.4 (0.8) | *P*=.51 | 0.15  (-0.15 to 0.44) |
|  | 10. Perceived adequacy of technical support | 4.1 (0.9) | 3.2 (1.0) | *P*=.09 | 0.42  (0.12 to 0.68) |
| *Workload* | 11. Perceived workload | 4.2 (1.1) | 3.8 (0.7) | *P*=.34 | 0.27  (-0.06 to 0.57) |
|  | 12. Perceived work-related stress | 1.9 (1.1) | 2.9 (1.5) | *P*=.23 | -0.35  (-0.62 to -0.03) |
| *Overall evaluation* | 13. Recommendation of the HaH model | 4.9 (0.4) | 4.6 (0.6) | *P*=.51 | 0.18  (-0.09 to 0.43) |
|  | 14. Overall satisfaction | 4.7 (0.9) | 4.6 (0.6) | *P*=.51 | 0.15  (-0.11 to 0.40) |

Table S3. Dunn’s post hoc pairwise comparisons by professional group. Multiplicity-adjusted *P* values are reported for pairwise comparisons following significant Kruskal–Wallis tests. GraphPad Prism (v.8.0.1) was used to calculate the multiplicity-adjusted *P* values; no additional multiplicity correction was applied. Statistical significance was defined as adjusted *P*<.05. Adjusted *P* values <.05 are shown in bold.

| Item | Physicians vs nurses, *adjusted* *P* value | Physicians vs nursing assistants,  *adjusted P* value | Nurses vs nursing assistants,  *adjusted P* value |
| --- | --- | --- | --- |
| 4. Perceived communication effectiveness with patients | ***P*=.035** | *P*>.99 | ***P*=.02** |
| 6. Perceived promotion of patient autonomy | ***P*=.001** | *P*=.88 | *P*=.11 |
| 7. Perceived ease of technology use | ***P*=.02** | *P*>.99 | *P*=.42 |
| 8. Perceived facilitation of work processes | ***P*=.02** | *P*>.99 | *P*=.18 |
| 9. Perceived contribution of technology to quality of care | ***P*=.001** | *P*>.99 | ***P*=.01** |
| 11. Perceived workload | ***P*=.03** | ***P*=.01** | *P*>.99 |
| 13. Recommendation of the HaH model | ***P*=.003** | *P*>.99 | ***P*=.005** |

Table S4. Mean (SD) and median [IQR] scores for items related to interaction with technology, stratified by training status (“Yes”, n = 22; “Partially”, n = 6; “No”, n = 2).

| Conceptual category | Item | Yes, mean (SD) | Partially, mean (SD) | No, mean (SD) | Yes, median [IQR] | Partially, median [IQR] | No, median [IQR] |
| --- | --- | --- | --- | --- | --- | --- | --- |
| *Interaction with technology* | 7. Perceived ease of technology use | 4.1  (0.7) | 3.3  (1.4) | 3.5  (0.7) | 4  [4-5] | 3.5  [2.5-4.25] | 3.5  [3-4] |
|  | 8. Perceived facilitation of work processes | 4.3  (0.7) | 4.3  (0.5) | 4  (0) | 4  [4-5] | 4  [4-5] | 4  [4-4] |
|  | 9. Perceived contribution of technology to quality of care | 4.6  (0.7) | 4.2  (0.7) | 3.5  (0.7) | 5  [4-5] | 4  [3.75-5] | 3.5  [3-4] |
|  | 10. Perceived adequacy of technical support | 4.0  (0.7) | 2.8  (1.2) | 2.0  (1.4) | 4  [3-4.25] | 3  [1.75-4] | 2  [1-3] |

Table S5. Optional open-ended comments from survey participants (n=6).

|  | Participants’ responses |
| --- | --- |
| 1 | **“**It would make the professionals’ work easier if the hospital’s internal network were faster.**”** |
| 2 | **“**Primary caregivers may become overwhelmed because of the number of tasks involved.**”** |
| 3 | **“**It is important not to rush when admitting patients and to take the time to explain everything properly.**”** |
| 4 | **“**Improvements need to be made to the electronic platform, such as adding a “seen” check, enabling direct inclusion of patients into the electronic clinical record system, and developing a specific program for the dashboards. Training sessions should be provided on the new processes (hematology, myeloma, etc.) within the platform, since doubts often arise in the afternoons about how to open these processes.**”** |
| 5 | **“**Nursing assistants, who are responsible for managing the electronic platform, do not have a protocol explaining how certain functions work.**”** |
| 6 | **“**We need more powerful equipment and better technology overall to ensure faster connections with fewer interruptions due to connectivity issues or any other cause.**”** |
